# Noise & mottle suppression methods for cumulative Cherenkov images of radiation therapy delivery

**DOI:** 10.1088/1361-6560/ad8c93

**Published:** 2024-11-12

**Authors:** Jeremy E Hallett, Petr Bruza, Michael Jermyn, Ke Li, Brian W Pogue

**Affiliations:** 1 Department of Medical Physics, University of Wisconsin-Madison, Madison, WI, United States of America; 2 Thayer School of Engineering at Dartmouth, Hanover, NH, United States of America; 3 DoseOptics LLC, Lebanon, NH, United States of America

**Keywords:** Cherenkov, denoising, total variation, block matching, non-local means, adaptive-trimmed mean, bilateral

## Abstract

*Purpose.* Cherenkov imaging during radiotherapy provides a real time visualization of beam delivery on patient tissue, which can be used dynamically for incident detection or to review a summary of the delivered surface signal for treatment verification. Very few photons form the images, and one limitation is that the noise level per frame can be quite high, and mottle in the cumulative processed images can cause mild overall noise. This work focused on removing or suppressing noise via image postprocessing. *Approach.* Images were analyzed for peak-signal-to-noise and spatial frequencies present, and several established noise/mottle reduction algorithms were chosen based upon these observations. These included total variation minimization (TV-L1), non-local means filter (NLM), block-matching 3D (BM3D), alpha (adaptive) trimmed mean (ATM), and bilateral filtering. Each were applied to images acquired using a BeamSite camera (DoseOptics) imaged signal from 6x photons from a TrueBeam linac delivering dose at 600 MU min^−1^ incident on an anthropomorphic phantom and tissue slab phantom in various configurations and beam angles. The standard denoised images were tested for PSNR, noise power spectrum (NPS) and image sharpness. *Results.* The average peak-signal-to-noise ratio (PSNR) increase was 17.4% for TV-L1. NLM denoising increased the average PSNR by 19.1%, BM3D processing increased it by12.1% and the bilateral filter increased the average PSNR by 19.0%. Lastly, the ATM filter resulted in the lowest average PSNR increase of 10.9%. Of all of these, the NLM and bilateral filters produced improved edge sharpness with, generally, the lowest NPS curve. *Conclusion.* For cumulative image Cherenkov data, NLM and the bilateral filter yielded optimal denoising with the TV-L1 algorithm giving comparable results. Single video frame Cherenkov images exhibit much higher noise levels compared to cumulative images. Noise suppression algorithms for these frame rates will likely be a different processing pipeline involving these filters incorporated with machine learning.

## 1. Introduction

**Figure 1.**
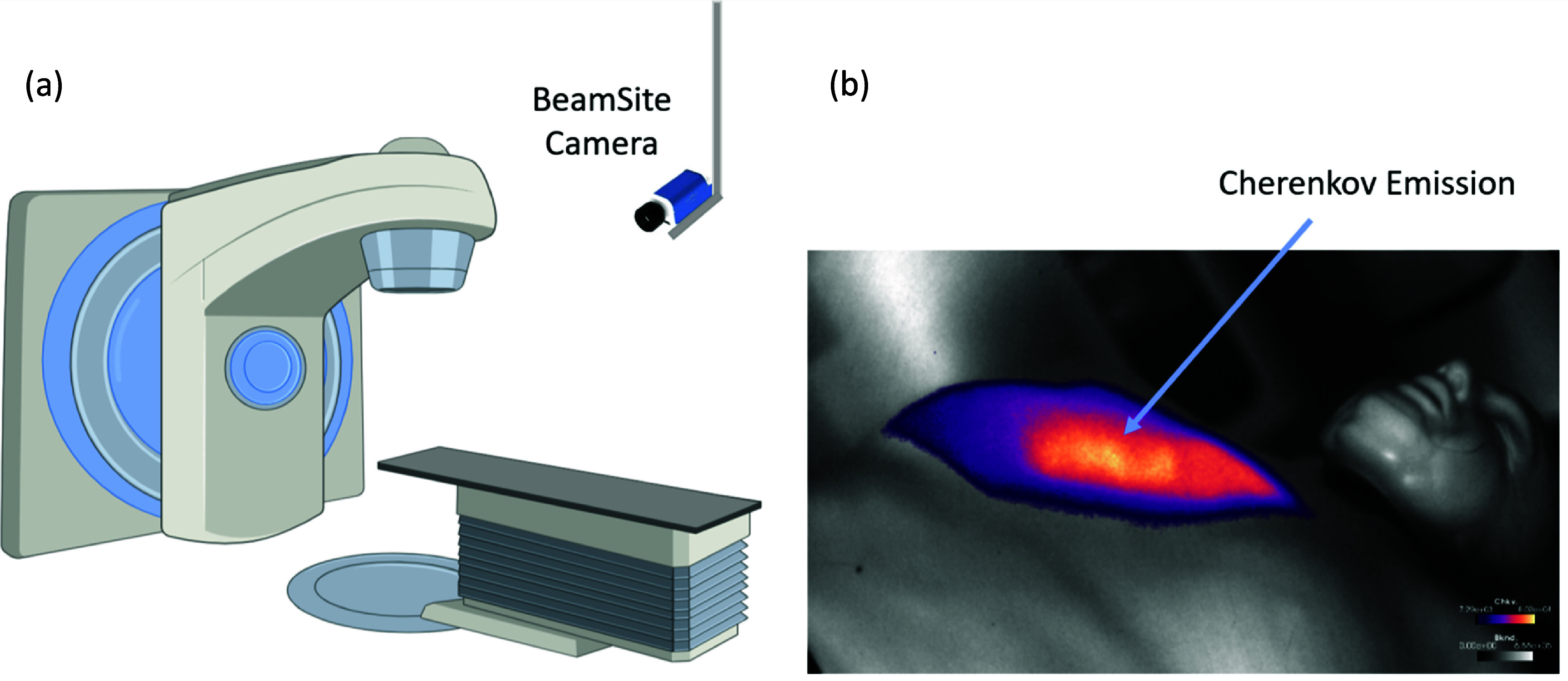
(a) The BeamSite camera is mounted to the ceiling and directed towards the isocenter of the linac. (b) Example of a cumulative Cherenkov image, from a 15 × 15 cm^2^, 6 MV linac beam, incident upon the right breast of a body phantom, delivering 200 MU in a single beam.

Image noise is an inherent part of all medical images, but those based upon low count statistics can be confounded the most by a mix of statistical noise and processing artifacts. There are a vast number of algorithms and reconstruction techniques that have been developed to minimize these effects. One imaging technique on which many of these algorithms have yet to be tested is relative surface dose map imaging with Cherenkov radiation. This radiation is produced in a dielectric medium when charged particles move faster than the speed that light travels inside that medium (Cherenkov 1934). When a patient is irradiated, the accelerated secondary electrons in the patient temporarily disturb the polarity of the medium and produce Cherenkov light which gets partially attenuated as it exits the patient’s tissue (Snyder *et al* 2018). This emitted light can be detected with a time-gated camera which acquires signal just during the pulses of the linac, removing background light (Glaser *et al* 2012). The camera is usually mounted to the ceiling as demonstrated in figure 1 (Jarvis *et al* 2021).

These images have many applications in the clinical setting, such as providing a surrogate image of dose delivered to the skin, or surface dose distribution (Jarvis *et al* 2021, Alexander *et al* 2023). Furthermore, Cherenkov light can be used as an image-guided radiation therapy tool for verifying dose delivery and detecting improper dose deposition incidents (Chen *et al* 2022). Although these research areas have experienced success, the images themselves suffer from large amounts of noise and subsequent post processing artifacts from temporal and spatial filtering.

Cherenkov imaging is an inherently noisy process due to the low number of photons detected. The current processing of the BeamSite camera performs both a temporal and median filter in addition to background subtraction to create the final image. Although these steps of the processing do an excellent job of removing much of the background light as well as some of the salt and pepper noise present in the unfiltered images, they still allow a large level of noise into the image sequence. The goal of this work is to apply various image denoising algorithms used in other medical imaging applications to the noisy Cherenkov images to survey the possible improvements such a denoising approach could offer. This analysis implemented a Total Variation denoising algorithm with an L1 norm (TV-L1), a Non-Local Means denoising algorithm (NLM), an Alpha (adaptive) Trimmed Mean filter (ATM), a bilateral filter and a formulation of the Block-Matching 3D-transform denoising method (BM3D).

There are two means of viewing Cherenkov data. One could focus on each frame of the Cherenkov video which is the case when viewing the Cherenkov data emitted from a patient in real-time. This viewing method is necessary if there is a lot of patient movement throughout the treatment and also useful for modulated treatments where the beam is changing throughout the treatment. However, if the patient remains relatively still one could sum all the frames together to produce a cumulative image. Because of the principles of counting statistics, the cumulative image experiences an increase in signal to noise ratio (SNR) relative to the single-frame images. Despite the increase in image quality, the summed image still experiences extensive blotches of noise from the summed noise signal. This inhomogeneity can be improved with noise reduction algorithms.

This research looked to determine which of the five algorithms implemented provides the greatest improvement in image quality. The algorithm that performs the best with cumulative images is likely to be the most suited for implementation in a series of processing steps for denoising single frame images.

It should be noted that many modern denoising approaches incorporate machine learning in the filtering pipeline. However, these filters often focus on supervised learning which require large data sets with ground truth images to guide the learning process. Such data sets are difficult to obtain in the medical imaging regime. This work is motivated by this obstacle as classical denoising algorithms can improve cumulative image quality to act as ground truth data for single frame machine learning models. This topic is discussed further in the discussions section of this article.

## 2. Methods

### 2.1. Experimental setup

The Cherenkov images used in this analysis were acquired using a BeamSite research Cherenkov camera (DoseOptics LLC, Lebanon NH) equipped with a Nikon 50 mm lens. Both the temporal and spatial median filters which smooth images using the median value across several frames or pixels were implemented with all other acquisition settings in the cameras data acquisition software. The radiation source in this study was a Varian TruBeam linear accelerator set to deliver an output of 9000MU with an x-ray beam energy of 6 MV at a dose rate of 600 MU min^−1^. These long exposures produced close to 10 000 frames per acquisition which were summed to produce a low noise ‘ground truth’ estimate for comparison with noisier images. Various levels of noise were obtained by lowering the number of frames accumulated in each noisy image to match the desired linac output in MU. The radiation field was incident on an anthropomorphic silicone body phantom and a silicone tissue slab phantom arranged in several configurations and beam angles to produce various images for algorithm testing. Silicone is an excellent approximation of human tissue as the typical density of many prosthetic silicone products (1.07 g cm^−3^) (Smooth-On) closely matches that of soft tissue (1.04 g cm^−3^). Additionally, the effective atomic number of generic silicone is 10.65 (Aldosary *et al* 2022) which is similar to that of soft tissue (7.35). For the majority of the energy range of 6 MV linac photons interacting with both soft tissue and silicone, the dominant interaction is Compton scatter off of electrons which is roughly independent of the atomic number of the material leading to similar interactions of radiation with both media. These phantoms consisted of silicone mixed with skin tone pigment which has been documented in other work for use in making tissue mimicking optical phantoms (Zhao *et al* 2023). The BeamSite camera was mounted to the ceiling and angled to face the isocenter of the linac as shown in figure 1. The phantom was roughly located at the isocenter for each acquisition. All post-processing was carried out with MATLAB version R2023b run on a ThinkPad laptop with a 2.4 GHz intel processor and 32GB of RAM.

### 2.2. Total variation L1 (TV-L1) processing

TV-L1 denoising is a powerful method that produces a smoother image by minimizing the value of a specific cost function that seeks to balance less variation with a denoised imaged similar to the input (Rudin *et al* 1992, Roscani *et al* 2020). The TV-L1 formulation listed in Lourakis (2023) was applied to the cumulative Cherenkov images. The cost function is split into two terms. The first term minimizes the variation of pixel intensity between sequential pixels in the *x* and *y*-axes. The second term is the regularization term and is defined in a way that maintains the sharp features of the image

\begin{align*}\arg \;{\text{min}}_{{{I}} \in \mathbb{R}}N{\left\| {\nabla {\boldsymbol{I}}} \right\|_2} + \lambda {\left\| {{\boldsymbol{I}} - {\boldsymbol{G}}} \right\|_1}.\end{align*}

In this formulation, **I** is the denoised image, **G** is the original image, and $\nabla I$ takes the gradient of the denoised image along both *x* and *y* axes (Lourakis, 2023). This cost function is minimized using a primal-dual algorithm (Dantzig *et al* 1956). The term $\lambda $ is the regularization coefficient and can be varied to determine the level of denoising aggression. This weighting factor determines the priority given to the regularization term relative to the first term. The greater the $\lambda $ value, the more the algorithm attempts to minimize the variation between **I** and **G**, essentially recreating the same noisy image. The lower $\lambda $ is set, however, the harsher the algorithm smooths out the image. A balance must be met to obtain adequate denoising without imposing artifacts from an over-weighted variation term. The algorithm accepts the noisy image, $\lambda $, and the number of iterations as inputs. For this analysis, 100 iterations was selected with $\lambda $ values assessed from $\lambda = 3.5$ to $\lambda = 0.01$ based on the PSNR improvement and balanced by the need to retain high spatial resolution features of the image.

### 2.3. Non-local means (NLM) processing

The NLM method (Wu, 2023) takes advantage of repeated structures throughout an image to help eliminate random noise while preserving the important details (Darbon, 2008). The original NLM implementation developed by Buades *et al* samples patches of pixels from the noisy image within a specified spatial window and performs a weighted average comparing each patch to a patch targeted for denoising located at the window center. The weights for each patch are determined by the patch’s similarity to the patch being denoised.

\begin{align*}\begin{array}{*{20}{c}} {u\left( s \right) = \frac{1}{{Z\left( s \right)}}\mathop \sum \limits_{t \in \mathcal{M}\left( s \right)} w\left( {s,t} \right)v\left( t \right)} \end{array}\end{align*}

*u*(*s*) is the denoised patch centered at location *s, w*(*s,t*) is the weight determined by comparing a patch at site t inside the denoising window to the target patch centered on *s, v*(*t*) represents various patches of the original noisy image, *Z*(*s*) is a normalization factor, and $\mathcal{M}\left( s \right)$ is a specified search window. $\mathcal{M}\left( s \right)$ is centered on the target pixel to limit the number of comparison patches. The window dimensions can be adjusted along with the patch size to acquire varying results. In the original approach, the weights were calculated as the inverse exponential of the sum of square distances multiplied by a Gaussian or constant (Buades *et al* 2005).

\begin{align*}\begin{array}{*{20}{c}} {w\left( {s,t} \right) = {e^{\frac{{ - \left( {\mathop \sum \nolimits_{\delta \in {{\Delta }}} G\left( \delta \right){{\left( {v\left( {s + \delta } \right) - v\left( {t + \delta } \right)} \right)}^2}} \right)}}{{{\sigma ^2}}}}}} \end{array}\end{align*}

Δ describes the patch dimensions, $G\left( \delta \right)$ is the Gaussian function, and *σ* is a parameter used to set the denoising aggressiveness. However, this NLM approach suffers from long computation times which led to the development of a fast NLM algorithm by Darbon *et al* This fast approach uses the same weighted average implementation but vectorizes the algorithm that determines the weights to decrease the computation time.

The fast NLM approach achieves better speed by vectorizing the original formulation. Creating an integral image of the square differences

\begin{align*}\begin{array}{*{20}{c}} {{{\boldsymbol{S}}_{\boldsymbol{d}}}\left( l \right) = \mathop \sum \limits_{k = 0}^l {{\left( {{\boldsymbol{v}}\left( k \right) - {\boldsymbol{v}}\left( {k + d} \right)} \right)}^2},} \end{array}\end{align*}

where $d$ is a shift of the padded noisy image **v** in the *x* and *y* direction ($d = t - s$), the fast algorithm re-parametrizes the weight equation to produce a simpler expression

\begin{align*}\begin{array}{*{20}{c}} {w\left( {s,t} \right) = {\text{exp}}\left( { - \left( {{{\boldsymbol{S}}_{\boldsymbol{d}}}\left( {s + P} \right) - {{\boldsymbol{S}}_{\boldsymbol{d}}}\left( {s - P} \right)} \right)/{\sigma ^2}} \right)} \end{array}\end{align*}

*P* describes the *x* and *y* half dimensions of the patch size set by the user. In this approach, every patch is analyzed in parallel. The algorithm works by computing an ${S_d}$ matrix for various values of l and a given shift d within the window dimensions. The algorithm then uses equation (5) to compute a weight matrix for each shift. The noisy, shifted image is multiplied by the appropriate weighting matrix and added to the final, denoised image similar to equation (2) (Darbon 2008).

The fast algorithm was chosen for this analysis, requiring the noisy image, the size of the search window and patches, as well as the σ defined above as inputs. Just like with the total variation denoising approach, one can adjust the parameters to obtain the best filtering results while avoiding significant image artifacts. The fast NLM algorithm was applied to Cherenkov images and many values of σ from 0 to 7000 were assessed for each cumulative image to achieve the best improvement in PSNR. The search window was set to a 20 × 20 pixel search window and a 4 × 4 pixel patch size.

### 2.4. Block-matching 3D (BM3D) processing

The BM3D-transform filter was developed based on the work of Makinen *et al* (2020). The specific version used was version 3.0.9 released in 2021 by the Tampere University of Technology. This algorithm is more complicated than the previous filters and is composed of the following steps. The algorithm starts with block matching where a reference block is compared against all noisy blocks that exist within a local search region, much like the NLM approach. Blocks are then matched based on their similarity to the reference block to produce a list of M noisy blocks. The matching criteria is based on equation (6)

\begin{align*}\begin{array}{*{20}{c}} {{L_{{x_R}\left( {{x_j}} \right)}} = \left\| {{z_{{x_R}}} - {z_{{x_j}}}} \right\|_2^2 - 2\gamma \mathop \sum \limits_{i = 1}^N \nu _{i,2}^{{x_R},{x_j}}} \end{array}.\end{align*}

**Figure 2.**
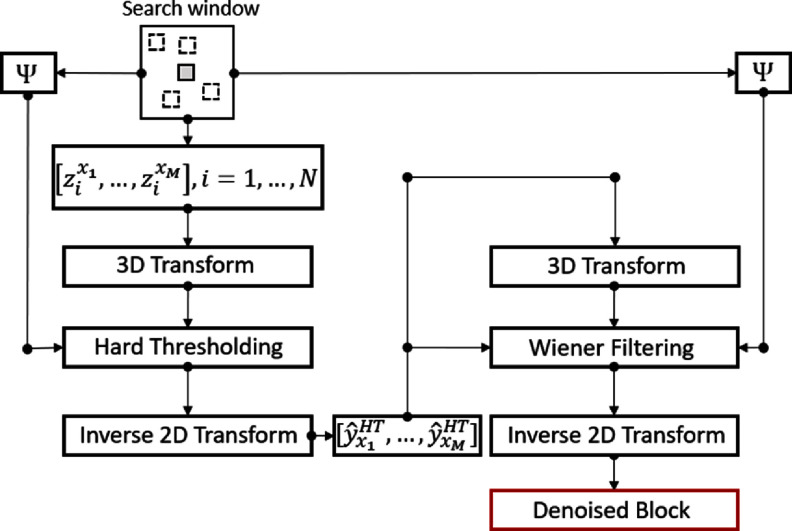
Processes of the BM3D algorithm. The solid, gray block in the top search window represents the reference block and the dotted squares are the matched blocks. ${{\Psi }}$ is the power spectral density (PSD) of the noise in the original image. Details are in Makinen *et al* (2020).

The reference block is defined as ${z_{{x_R}}}$while the block being compared is ${z_{{x_j}}}$. The second factor is subtracted to remove the positive bias produced by the image noise for a roughly noise free comparison of the two blocks for proper sorting. $\gamma $ is a weighting factor that is often set to 3 for the algorithm’s first step, but set to 0 for the subsequent Weiner filtering step. ${x_R}$ and ${x_j}$ are the reference and comparison block coordinates. Additionally, *N* is the number of pixels inside each block and $\nu _{i,2}^{{x_R},{x_j}}$ describes the variance of the 3-dimensional transform of the reference and comparison blocks. Once the blocks are matched, the same 3D transform mentioned above is applied to the group of blocks. First, a 2-dimensional transform is applied to each block and denoted by $s_i^{{x_j}} = {z_{{x_j}}},b_i^{2D}$, where $b_i^{2D}$ describes the *i*th basis function of the transform. A 1-dimensional transform is then applied to the set of *M* transformed blocks $\left[ {s_i^{{x_1}}, \ldots ,s_i^{{x_M}}} \right],i = 1, \ldots ,N$. This results in a set of 3D transformed blocks denoted as $\left\{ {s_{i,j}^{{x_1}, \ldots ,{x_M}},i = 1, \ldots ,N,j = 1, \ldots ,M} \right\}$.

After performing this 3-dimensional transform, the transformed blocks are subjected to a hard thresholding step where the corresponding shrinkage attenuation factor ${\alpha _{i,j}}$ is set to either 0 or 1 depending on hard threshold defined below

\begin{align*}\begin{array}{*{20}{l}} {\alpha _{i,j}^{HT} = \left\{ {\begin{array}{*{20}{l}} {1{ if }\left| {s_{i,j}^{{x_1}, \ldots ,{x_M}}} \right| \unicode{x2A7E} \sqrt {\nu _{i,j}^{{x_1},\ldots,{x_M}}} \lambda } \\ {0{\,{otherwise}},{\text{ }}} \end{array}} \right.} \end{array}\end{align*}

$\nu _{i,j}^{{x_1},\ldots,{x_M}}$ is the variance of the transformed blocks and can be estimated using the power spectral density (${\text{PSD}}$) which is a measure of the image noise where signal power is a function of frequency. of the noise of the image being filtered

\begin{align*}\begin{array}{*{20}{c}} {\nu _{i,j}^{{x_1},\ldots,{x_M}} = {{\left| {\left| {{{\left| x \right|}^{ - 2}}{{\Psi }}{{\left| {FT\left( {b_i^{dD}} \right)} \right|}^2}{{\left| {FT\left( {\tilde b_j^{NL}} \right)} \right|}^2}} \right|} \right|}_1}} \end{array}\end{align*}

${{\Psi }}$ is the PSD of the noisy image while $FT$ denotes the Fourier Transform. Essentially, the PSD is a necessary input into the BM3D algorithm to properly denoise an image.

Once the attenuation factors are calculated, they are used combine the data into a denoised estimation

\begin{align*}\begin{array}{*{20}{c}} {{{\hat y}_{{x_j}}} = {Q^{dD}} \left\langle {\alpha_{i,j}^{{x_1}, \ldots ,{x_M}}s_{i,j}^{{x_1}, \ldots ,{x_M}},q_j^{NL}} \right\rangle } \end{array}\end{align*}

where ${Q^{dD}}$ is the inverse 2-dimensional transform applied previously and $q_j^{NL}$ is the *j*th basis function of this inverse transform. These estimations of each block get aggregated with weights set to give less importance to blocks with larger variance. The result is a denoised reference block contributing to a denoised image. Once this hard threshold filtering is complete, each step is repeated with a Wiener filter, a filter that eliminates high frequencies (low pass filter) with a cutoff dependent on the local image detail, taking the place of the hard thresholding step to calculate the shrinkage attenuation factors

\begin{align*}\begin{array}{*{20}{c}} {\alpha _{i,j}^{{\text{wiener}}} = \frac{{{{\left| {\left| {\langle \left[ {\hat y_{{x_1}}^{HT}, \ldots ,\hat y_{{x_M}}^{HT}} \right],b_i^{dD} \otimes b_j^{NL}\rangle } \right|} \right|}^2}}}{{{{\left| {\left| {\langle \left[ {\hat y_{{x_1}}^{HT}, \ldots ,\hat y_{{x_M}}^{HT}} \right],b_i^{dD} \otimes b_j^{NL}\rangle } \right|} \right|}^2} + {\mu ^2}\nu _{i,j}^{{x_1}, \ldots ,{x_M}}}}} \end{array}.\end{align*}

Equation (10) uses $\hat y_{\text{ }}^{HT}$ which is the denoised image from the hard thresholding step.

The paper by Mäkinen *et al* describes the entire process of the algorithm in detail, including various filters and steps neglected in this explanation (Makinen *et al* 2020).

The inputs for this algorithm are the noisy image and the expected PSD of the image noise. When this algorithm was applied to Cherenkov data, a PSD of Gaussian white noise with variance *σ*^2^ was assumed with σ varied between 0 and 10 000 to obtain the greatest increase in PSNR. Unfortunately, the actual PSD of Cherenkov image noise does not perfectly correlate to Gaussian white noise which likely contributed to the poor denoising performance discussed in the results of this paper. A deeper understanding of the noise pattern origins in Cherenkov images would likely improve the BM3D performance.

### 2.5. Alpha (adaptive) trimmed mean (ATM) processing

This algorithm was selected for its prevalent use in the field of noise suppression in CT reconstruction. The alpha (adaptive) trimmed mean filter (ATM) used in CT streak reduction was formulated by Jiang Hsieh while working for GE (Hsieh 1995). CT images often suffer from streak artifacts resulting from photon starvation when thicker regions of the patient with substantial bone or iodinated contrast material attenuate the x-ray beam (Hsieh 1995). Because the thickness of the patient varies depending on the path of the x-rays, some projections receive less signal than others. This signal variation is what produces the streaks. The original aim of this algorithm was to reduce the streaking artifacts present in CT images by adaptively filtering detector cells relative to their signal level. A one-dimensional filter was applied to each projection prior to filtered back projection. The code used in this analysis was developed based on Hsieh (1995) and adjusted for a two-dimensional case so that it could be applied to Cherenkov imaging.

Much like the case in CT, the trimmed mean filter used in this analysis was adaptive such that lower signal pixel values received more aggressive denoising than their higher signal counterparts. This was to ensure that the spatial resolution of the image was maintained while also smoothing the most cluttered regions. The trimmed mean filter itself is defined by a window size, *M*, and a trimming parameter specified as $\alpha $. Here, $\alpha $ describes the fraction of pixels trimmed from the ends of an array comprised of the pixel values within a specified window ordered from lowest to highest intensity. The domain of alpha goes from 0 to 0.5, while *M* is defined only for odd window sizes to accommodate a target pixel at its center. Mathematically, the trimmed mean filter can be defined by the following equation:

\begin{align*}\begin{array}{*{20}{c}} {{\boldsymbol{u}}{_j} = \mathop \sum \limits_i {\xi _i}{x_i}} \end{array}\end{align*}

where ${{\boldsymbol{u}}_j}$ is the denoised pixel j and ${x_i}$ describes the pixels in the sorted window [${x_1},{x_2},{x_3}, \ldots ,{x_{{M^2}}}$] going from lowest to highest intensity. ${\xi _i}$ is represented by the equation below:

\begin{align*}\begin{array}{*{20}{l}} {{\xi _i} = \left\{ {\begin{array}{*{20}{l}} {\frac{1}{{{M^2} - 2\alpha {M^2}}},{\text{ }}\alpha {M^2} &lt; i \unicode{x2A7D} {M^2} - \alpha {M^2}} \\ {0,{\,{ otherwise }}} \end{array}} \right.} \end{array}\end{align*}

where $\ldots $ represents the integer greater than or equal to whatever is inside the brackets. As $\alpha $ approaches 0, the filter simply performs an average over all the pixel values within the window and sets the target pixel equal to the resulting value. As $\alpha $ approaches 0.5, on the other hand, the filter turns into a median filter where all pixels are trimmed except for the pixel with the median intensity value (${x_{{M^2}/2}}$). In this case, the target pixel acquires the median value. The purpose of trimming the extreme values is to remove outlier (noisy) information when performing the averaging in image regions that should be homogenous (Hsieh 1998). This filter provides a balance between averaging over several pixels while selectively ignoring isolated spikes in intensity.

Because of the adaptive nature of noise related to the signal level in an image, both the *M* and $\alpha $ parameters were adapted relative to the pixel intensity.

\begin{align*} M &amp; = \frac{{2\beta \lambda \cdot \max \left( x \right)}}{{2\lambda \cdot \max \left( x \right) + \beta \cdot p\left( {x - \delta } \right)}} \end{align*}

\begin{align*} \alpha &amp; = \frac{x}{{\lambda \cdot \max \left( x \right)}} .\end{align*}

The function *p*() in equation (13) is defined as:

\begin{align*}p\left( z \right) = \left\{ {\begin{array}{*{20}{c}} {z,{\text{ }}\,\,\,\,\,z &gt; 0} \\ {0,{\text{ }}\,\,\,\,\,z \unicode{x2A7D} 0} \end{array}} \right.\end{align*}

$\beta $, $\lambda $, and $\delta $ are parameters chosen to maximize the denoising capabilities of the filter, while Max(*x*) is the maximum pixel value in the image. $\beta $ denotes the maximum possible window size and was set to 31 pixels for this implementation. $\delta $ is a parameter employed to minimize internal camera noise noticeable at very low signal values. Any signal less than $\delta $ is given the maximum window size of $\beta $. $\delta $ was set equal to a pixel value of 5. Finally, $\lambda $ helps define the aggressiveness of the denoising. As $\lambda $ increases, the window size approaches $\beta $ for all pixel values and the trimming decreases. One consequence of this formulation is that one must set $\lambda \unicode{x2A7E} 2$ to ensure that $\alpha \unicode{x2A7D} 0.5$. Similar to the analysis conducted with the other algorithms, various $\lambda $ values were assessed between 2 and 14 and the PSNR measured to identify the best reduction in noise possible with the ATM approach each time the algorithm was applied.

### 2.6. Bilateral filter

As already stated, an ideal denoising filter for Cherenkov imaging will smooth out the noise pattern while maintaining sharp edge features and variable brightness within the Cherenkov signal related to the geometric position of the light emission. The bilateral image filter is specifically designed to maintain such features through the combination of range and domain filtering (Tomasi and Manduchi 1998).

For the denoising of homogenous pixel regions, a geometric closeness function can be used, performing a weighted average of local pixel intensity with the weights determined by how close each pixel is to the target pixel. This approach is termed domain filtering as it relies on the similarities of the domain values. However, such an algorithm is limited as it is unable to adequately preserve edges which get blurred due to the filtering. Blurring can be counteracted using a gray level similarity function. This approach is termed range filtering and performs a weighted average where the weights are calculated from local pixels with similar intensities (Tomasi and Manduchi 1998).

The algorithm is described by equation (16),

\begin{align*}\begin{array}{*{20}{c}} {{I_F}\left( p \right) = \frac{1}{W}\mathop \sum \limits_{q \in S} {G_{{\sigma _s}}}\left( {p,q} \right){G_{{\sigma _r}}}\left( {I\left( p \right),I\left( q \right)} \right)I\left( q \right)} \end{array}\end{align*}

where ${G_{{\sigma _s}}}\left( {p,q} \right)$ is the geometric closeness Gaussian function, ${G_{{\sigma _r}}}\left( {I\left( p \right),I\left( q \right)} \right)$ is a pixel intensity similarity function and *W* is a normalization factor (Shreyamsha Kumar 2013)

\begin{align*} {G_{{\sigma _s}}}\left( {p,q} \right) &amp; = {e^{ - \frac{{{{\left(p - q\right)}^2}}}{{2\sigma _s^2}}}} \end{align*}

\begin{align*} {G_{{\sigma _r}}}\left( {I\left( p \right),I\left( q \right)} \right) &amp; = {e^{ - \frac{{|I\left( p \right) - I\left( q \right){|^2}}}{{2\sigma _r^2}}}} \end{align*}

${\sigma _s}$ and ${\sigma _r}$ are parameters controlling the contributions of surrounding pixels to the target pixel p from the geometric and intensity perspective, respectively. Larger values for these parameters result in greater contributions. The MATLAB function imbilatfilt was employed which requires the noisy image as well as ${\sigma _s}$ and ${\sigma _r}$as inputs. Both ${\sigma _s}$ and ${\sigma _r}$ were varied to find the combination that provides an optimal PSNR where ${\sigma _s}$ was varied from 1 to 18 and ${\sigma _r}$ was varied from $100 \cdot {\text{v}}$ to $11\;000 \cdot {\text{v}}$ where *v* is the variance of a background patch of one of the analyzed images (image 3) (Tomasi and Manduchi 1998).

### 2.7. Data analysis

#### 2.7.1. Peak-signal-to-noise ratio (PSNR) as a metric of success

The PSNR of an image was calculated here as the log of the ratio between the maximum possible pixel signal for the given variable type and the root mean square error of the pixel values where x is the ‘ground truth’ image and *x*′ is the noisy or denoised image (Nadipally 2019)

\begin{align*}\begin{array}{*{20}{c}} {{\text{PSNR}}\left( {{{x^{^{\prime}}}},{{x}}} \right) = 20{\,\,\,{\log }_{10}}\left( {\frac{{\max \left( {{x}} \right)}}{{{\text{RMSE}}\left( {{{x^{\prime}}},{{x}}} \right)}}} \right)} \end{array}.\end{align*}

For each denoised image, the PSNR was calculated and compared to the PSNR produced for the noisy image. The maximal PSNR was used as one metric to determine the optimal parameters for the denoising algorithms. PSNR calculations were carried out for all image setups and filters with linac outputs of 25MU and 50MU. Additionally, curves of PSNR as a function of linac output were produced for images 3 and 5. Refer to figure 6 for the image numbers.

#### 2.7.2. Feature sharpness

**Figure 3.**
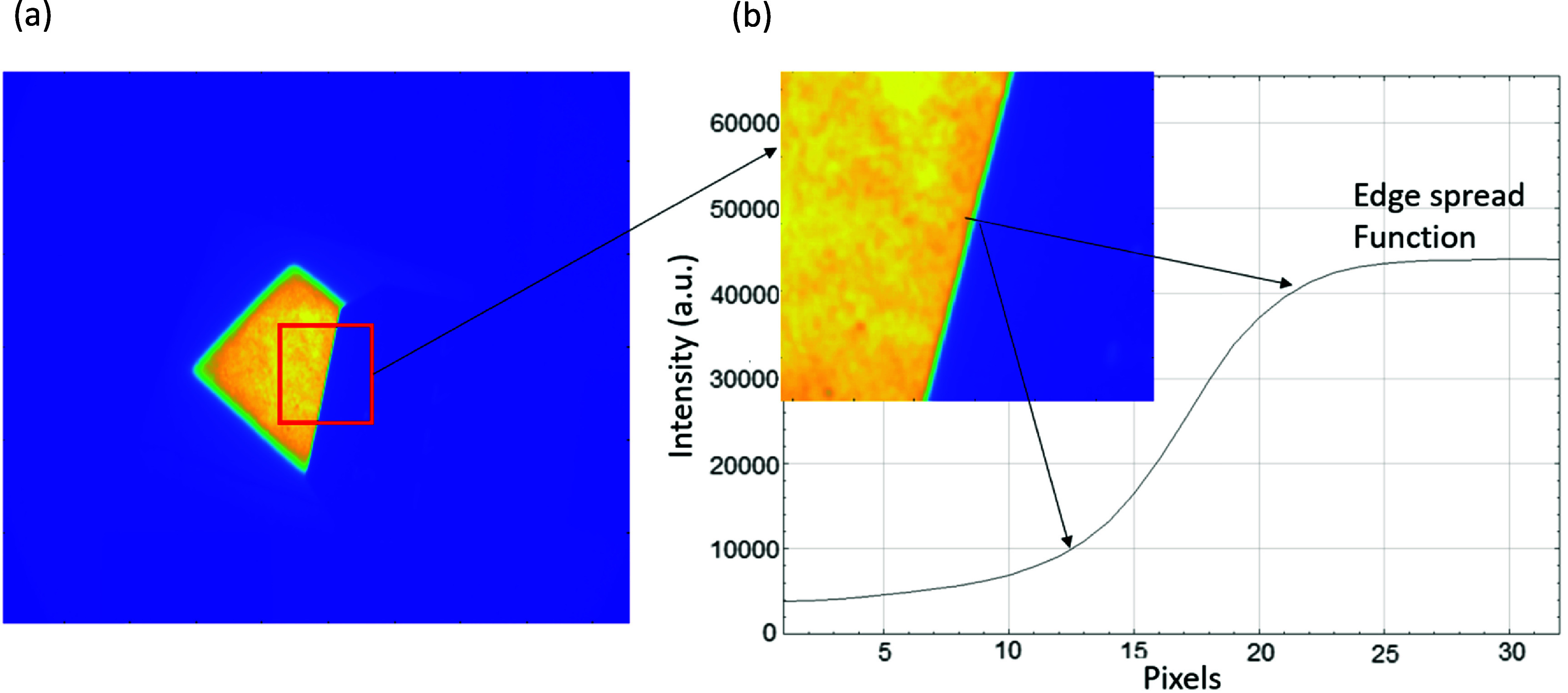
(a) Cherenkov image of a tissue phantom. The red box specifies the regions of the image used to assess image sharpness. (b) The right slanted edge of the unattenuated light in the Cherenkov image used for analysis of the image sharpness.

In addition to the analysis of the image PSNR, the edge sharpness of each image was also investigated. This analysis was necessary to determine if the filtered images suffered from extensive edge blurring. Two methods were implemented: the ten-to-ninety percent rise distance, and the modulation transfer function (MTF). As shown in figure 3, these metrics were measured on the slanted edge produced by blocking a region of Cherenkov light with a black plastic sheet.

**Figure 4.**
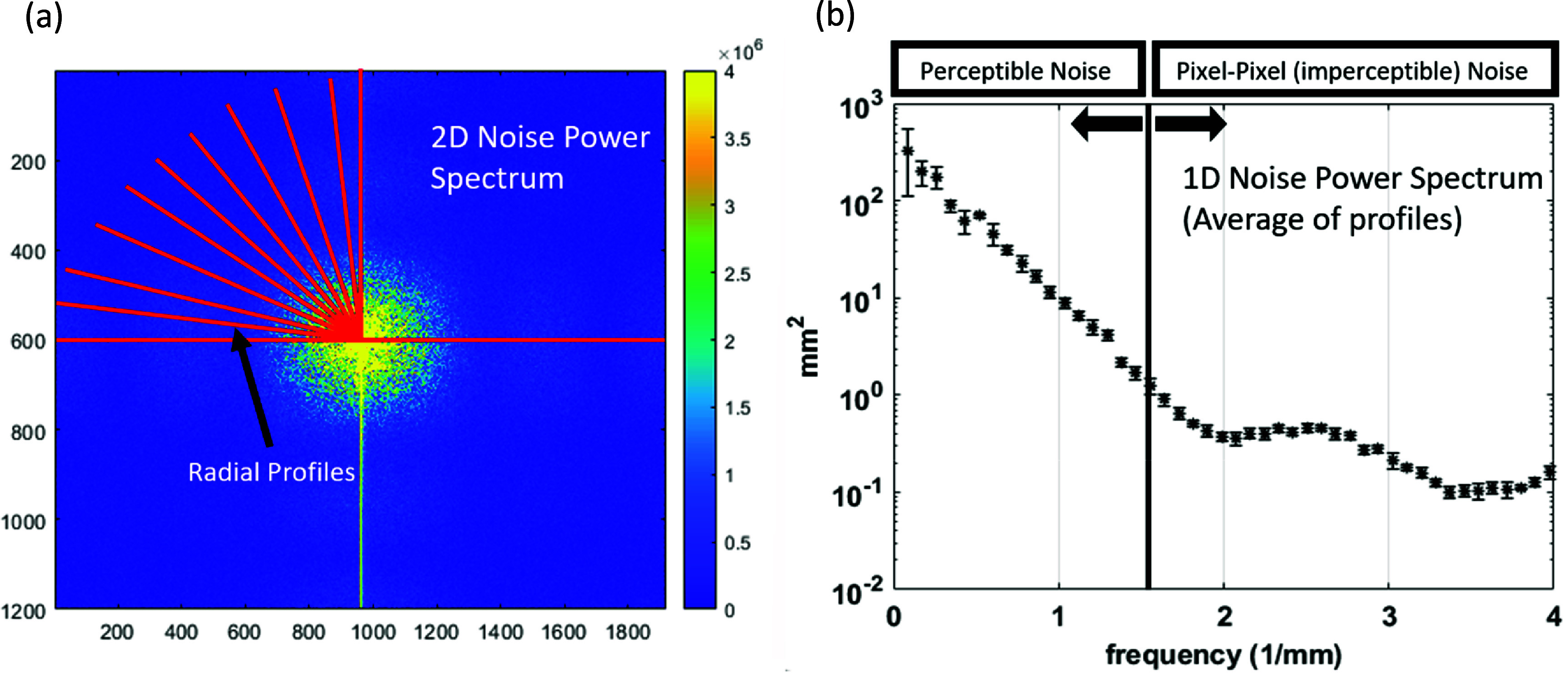
(a) Process of producing a radially average power spectrum by taking several radial profiles of the Fourier transform image. (b) Radially averaged power spectrum of the noisy Cherenkov image data. The percent difference plot is produced by comparing the filtered spectra to this spectrum.

The ten-to-ninety percent metric measures the sharpness by determining how many pixels in the edge spread function (ESF), which is effectively the profile of the edge, lie between the ten and ninety percent points of the rise height (Imatest n.d.). Additionally, the MTF is defined as the Fourier transform of the line spread function (LSF), which is defined as the derivative of the ESF and is measured in frequency (Masaoka *et al* 2014). The MTF is normalized to the value at zero frequency

\begin{align*} {\text{LSF}}\left( x \right) &amp; = \frac{{\text{d}}}{{{\text{d}}x}}{\text{ESF}}\left( x \right)\end{align*}

\begin{align*} {\text{MTF}}\left( \nu \right) &amp; = \left| {\mathop \int \limits_{ - \infty }^\infty {\text{LSF}}\left( x \right){e^{ - i2\pi \nu x}}{\text{d}}x} \right| .\end{align*}

The MTF metrics of sharpness used in this paper were MTF50 and MTF10. The MTF50 is the frequency where the MTF drops to fifty percent while the MTF10 is the frequency at ten percent where greater values correspond to higher resolution. These metric are used instead of the first zero crossing because the human eye is insensitive to spatial frequencies where the MTF is below ten percent (Imatest n.d.). The MTF was calculated using ImageJ and the slanted edge MTF plugin (Carles *et al* 2011).

#### 2.7.3. Radial frequency

To create a more complete understanding of the effect these post-processing algorithms have on the Cherenkov images, the noise power spectrum (NPS) was calculated. This analysis was conducted in a similar manner to the calculation described by Alexander *et al*, which was derived from the protocol outlined in the IEC 62 220-1 standard (Alexander *et al* 2020). The Cherenkov region of the silicone phantom images were segmented into several 100 × 100 pixel overlapping regions of interest (ROI) with the overlap determined by the geometric restrictions of the image. For each ROI, the square of the two-dimensional Fourier transform was calculated and then averaged with the other ROIs. This produced a two-dimensional NPS defined by the following equation

**Figure 5.**
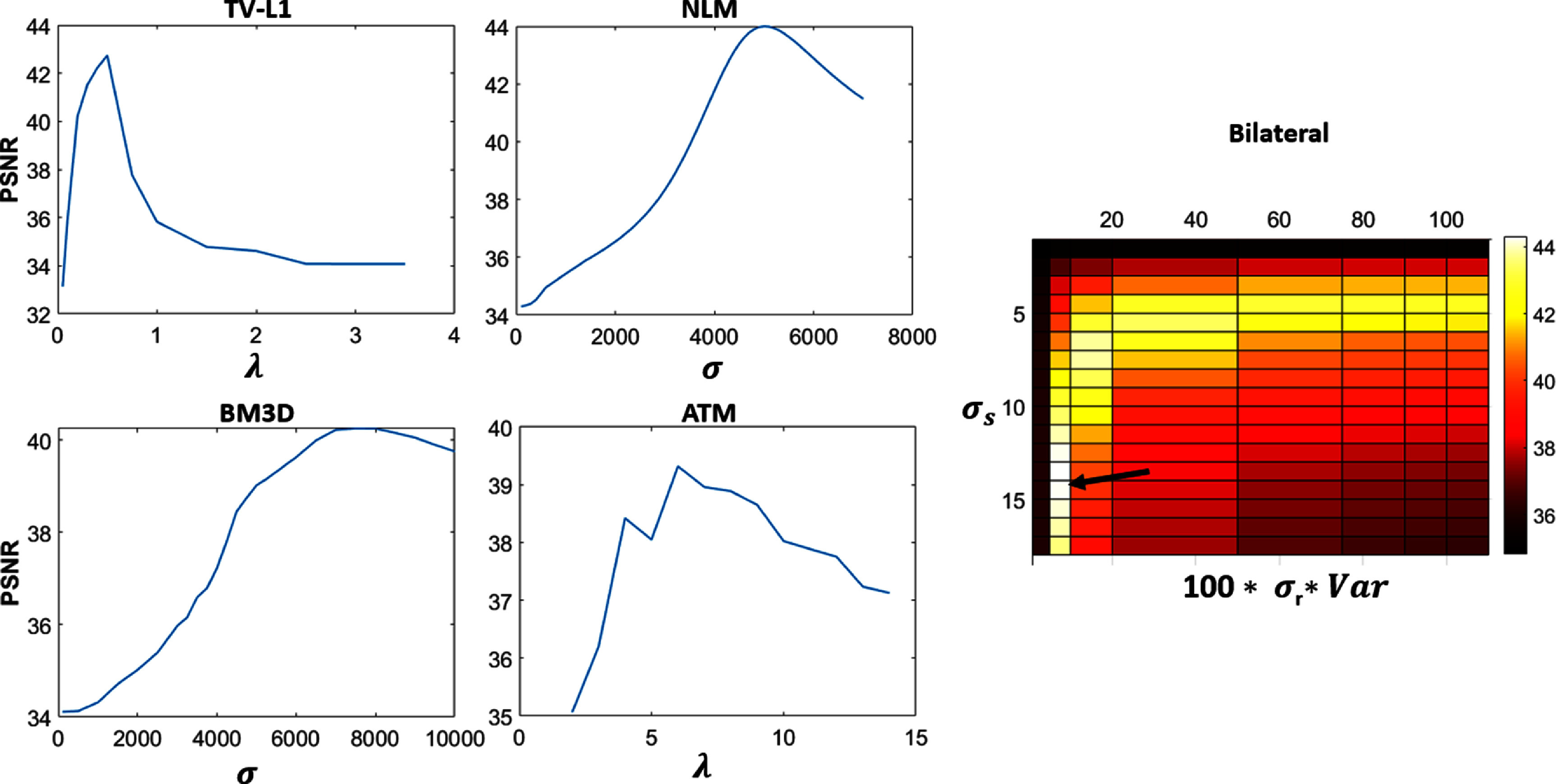
The PSNR was maximized for each denoised image by applying each algorithm several times with varying parameter values with the parameters producing the greatest PSNR being chosen. For the TV-L1, NLM, BM3D, and ATM algorithms, only $\lambda $ or $\sigma $ were varied, but for the bilateral filter, the optimization included both ${\sigma _s}$ and ${\sigma _r}$ requiring the most computations out of the five algorithms each time it was applied. These plots show an example of these optimizations for image 5 of the 25MU images.

\begin{align*}\begin{array}{*{20}{c}} {{\text{NPS}}\left( {{\nu _x},{\nu _y}} \right) = \frac{{\Delta x\Delta y}}{{M{N^2}}}\mathop \sum \limits_{{\text{m}} = 0}^{{\text{M}} - 1} {{\left| {\mathop \sum \limits_{i = 0}^{N - 1} \mathop \sum \limits_{j = 0}^{N - 1} I\left( {{x_j},{y_j}} \right){e^{ - 2\pi i\left( {{x_i}{\nu _x} + {y_j}{v_y}} \right)/{N^2}}}} \right|}^2}} \end{array}\end{align*}

where ${{\Delta }}x{{\Delta }}y$ is the physical size of each image pixel in the object plane, *N* is the dimension of each ROI (100 pixels) and *M* is the number of ROIs. The resulting matrix was reduced to a one-dimensional figure by averaging the radial profiles of the Fourier matrix to produce the radially averaged power spectrum (Ruzanski 2023). Figure 5(a) depicts the process of producing the radial power spectrum, while figure 5(b) demonstrates what the spectrum of the noisy data looks like.

## 3. Results

### 3.1. Peak signal-to-noise ratio

**Figure 6.**
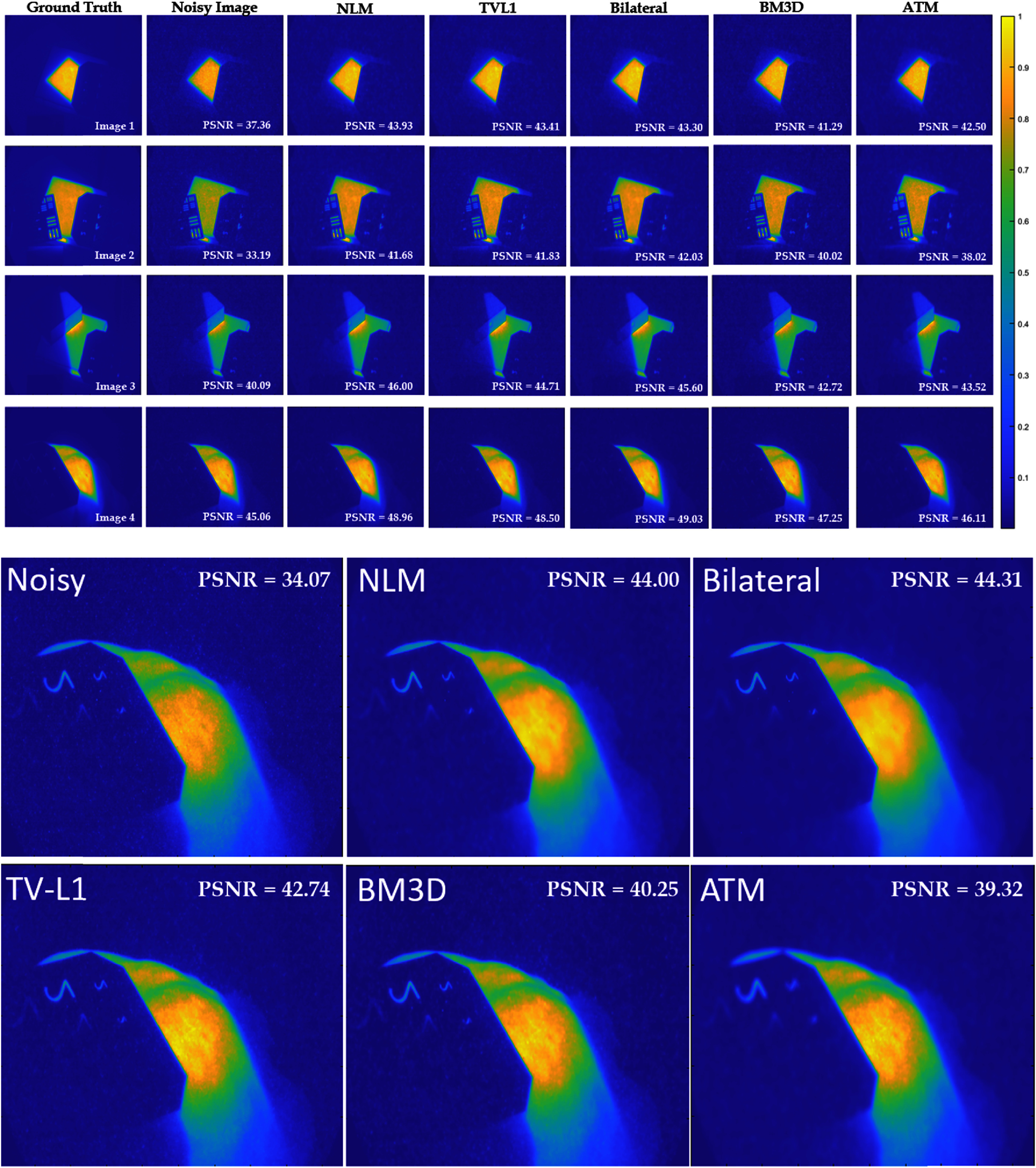
Five ground truth images were compared to noisy (25MU) and denoised images and the PSNR was calculated. NLM, TV-L1, and the bilateral filter all performed well with the NLM algorithm producing images with the largest PSNR on average. Both BM3D and ATM produced images with the lowest PSNR. Several images were considered to demonstrate the consistency of the results across images with different geometric, noise, and intensity features. Image 5 is given a larger display to demonstrate the effect of each filter compared to the noisy, unfiltered image.

Before filtering, the PSNR was calculated for the original noisy images. Each noisy Cherenkov image was then denoised using all five algorithms. Each algorithm was applied several times with various values of λ (for the TV-L1 and ATM implementations), σ (for NLM and BM3D), as well as ${\sigma _s}$ and ${\sigma _r}$ (for the bilateral filter). The maximum increase in PSNR was recorded for both 25MU and 50MU images and the algorithms were compared. The optimal performance of the TV-L1 algorithm applied to 25MU images was obtained when $\lambda = 0.3 - 0.5$. After filtering, the average PSNR increase was 17.4%. When the NLM filter was applied an optimal $\sigma $ was found between $4500-5500$ with an average PSNR increase of 19.1%. It should be noted that a truly optimal application of the NLM approach would extend the search window to the entire image, however, the window must be limited to a local neighborhood to avoid long computation times (Darbon 2008). The BM3D algorithm was applied with the optimized parameter $\sigma $ falling between 1500 and 3900 and a PSNR increase of 12.1%. Finally, the ATM filter had an optimal $\lambda $ range between 2 and 6 giving a PSNR increase of 10.9% while the bilateral filter saw a ${\sigma _s}$ range of 6–13 and a ${\sigma _r}$ optimized around the variance of the image Cherenkov signal multiplied by 100, producing an average PSNR increase of 19.0%. The ground truth, noisy, and denoised images are displayed in figure 6. An analysis of variance (ANOVA) test was conducted on this percent increase data resulting in a *p*-value of 0.3042.

50MU images showed a similar trend with the average PSNR increases being 2.92%, 8.70%, 7.82%, 6.29%, and 6.28% for ATM, NLM, bilateral, BM3D, and TV filters, respectively, with an ANOVA *p*-value of 0.2238. However, as the noise level is decreased, the BM3D algorithm becomes more comparable to NLM, TV, and the bilateral filter.

Although the *p*-values suggest that the PSNR increase was similar across algorithms, it is apparent that the NLM and bilateral algorithms supplied the greatest improvement with the TV-L1 algorithm producing comparable results when the noise level is high. The application of the BM3D and ATM approaches provide minimal improvements with the BM3D algorithm results degrading as the noise level increases. In fact, the BM3D code is known to decreases in effectiveness as the level of noise in the image increases (Fan *et al* 2019). This explains the Algorithm’s poor performance as Cherenkov images inherently exhibit high noise levels due to sparse photon collection.

**Figure 7.**
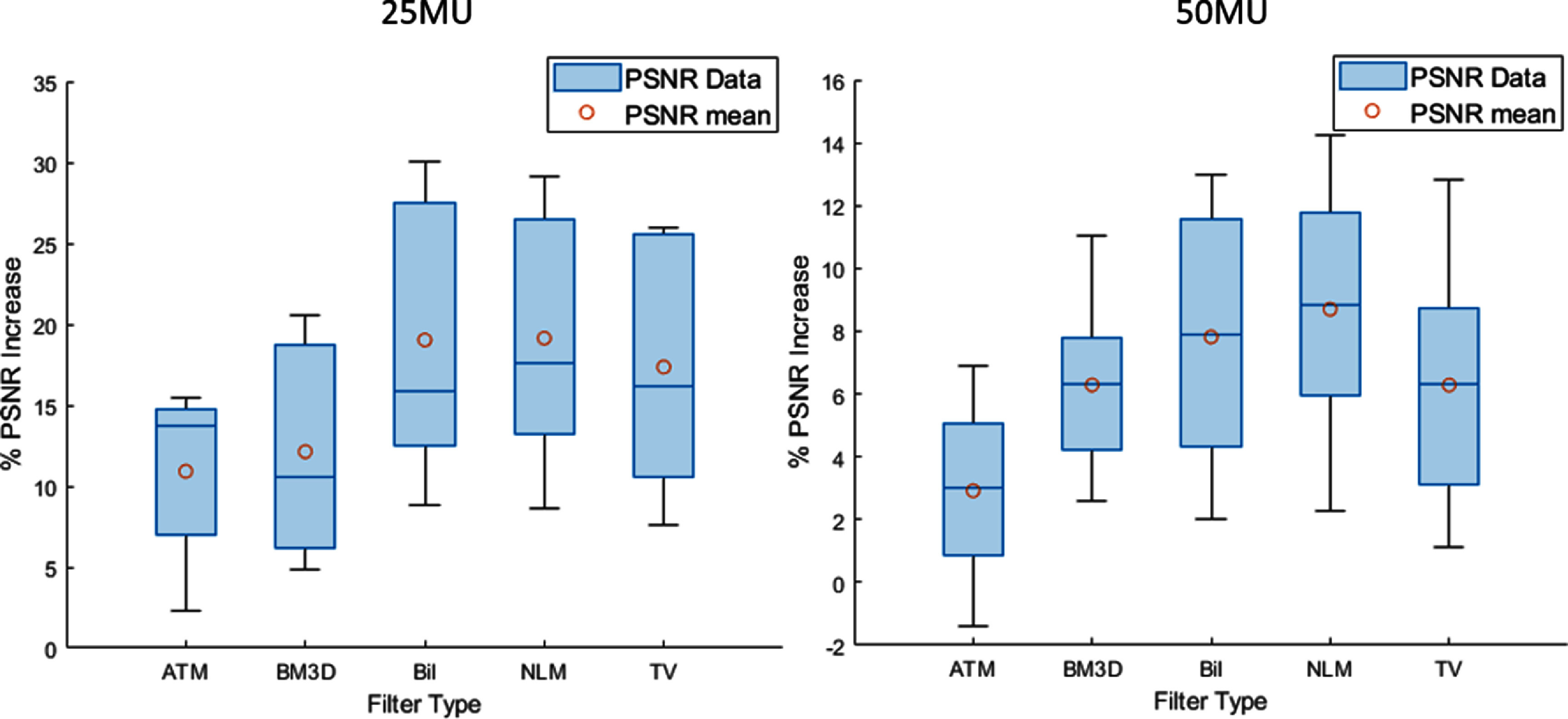
The percent PSNR increase for high noise (25MU) and moderate noise (50MU) images was calculated. Each set contained five images. This box plot shows the median (solid dark blue line) as well as the upper and lower quartile (upper and lower box boundaries) for the PSNR increase calculated for each algorithm. Additionally, the mean PSNR increase values are plotted.

**Figure 8.**
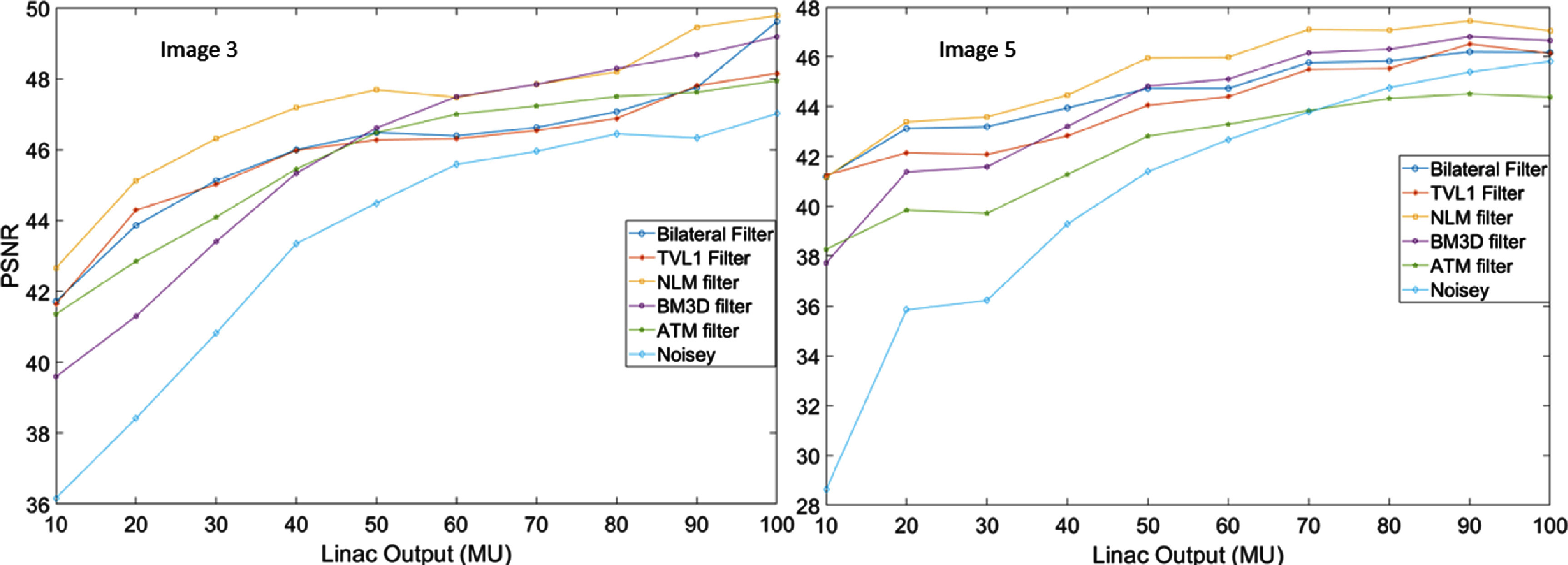
As the Cherenkov noise levels are increased (linac output decreased) each algorithm sees a different rate of degradation in their denoising effectiveness. The BM3D algorithm, for example, shows a large relative decrease in denoising ability as the noise is increased.

Figure 5 shows examples of the parameter optimization process. The PSNR was calculated as a function of the filtering parameters. Increasing the denoising aggressiveness past what was deemed optimal either results in artifacts or non-optimal image blurring. Figure 6 shows the ‘ground truth’ images side-by-side with the noisy images and the filtered images and figure 7 shows the percent increase of PSNR for each image after implementing each algorithm. Finally, figure 8 shows the PSNR of each algorithm as the noise level changes for images 5 and 3.

As seen in figure 8, the denoising ability of each algorithm as a function of noise level was demonstrated through the calculation of the optimal PSNR for images 3 and 5 across a wide range of linac output values (see figure 6 for visualization of images 3 and 5). For both images, the BM3D algorithm shows the same trend of degrading with higher noise while the ATM filter demonstrates excellent denoising at lower noise levels for image 3 but a PSNR less than the original noisy data for image 5. Based only on the PSNR, the TV, NLM, and bilateral filters are the optimal choices when denoising images with linac outputs between roughly 10 and 40 MU.

### 3.2. Feature sharpness

**Figure 9.**
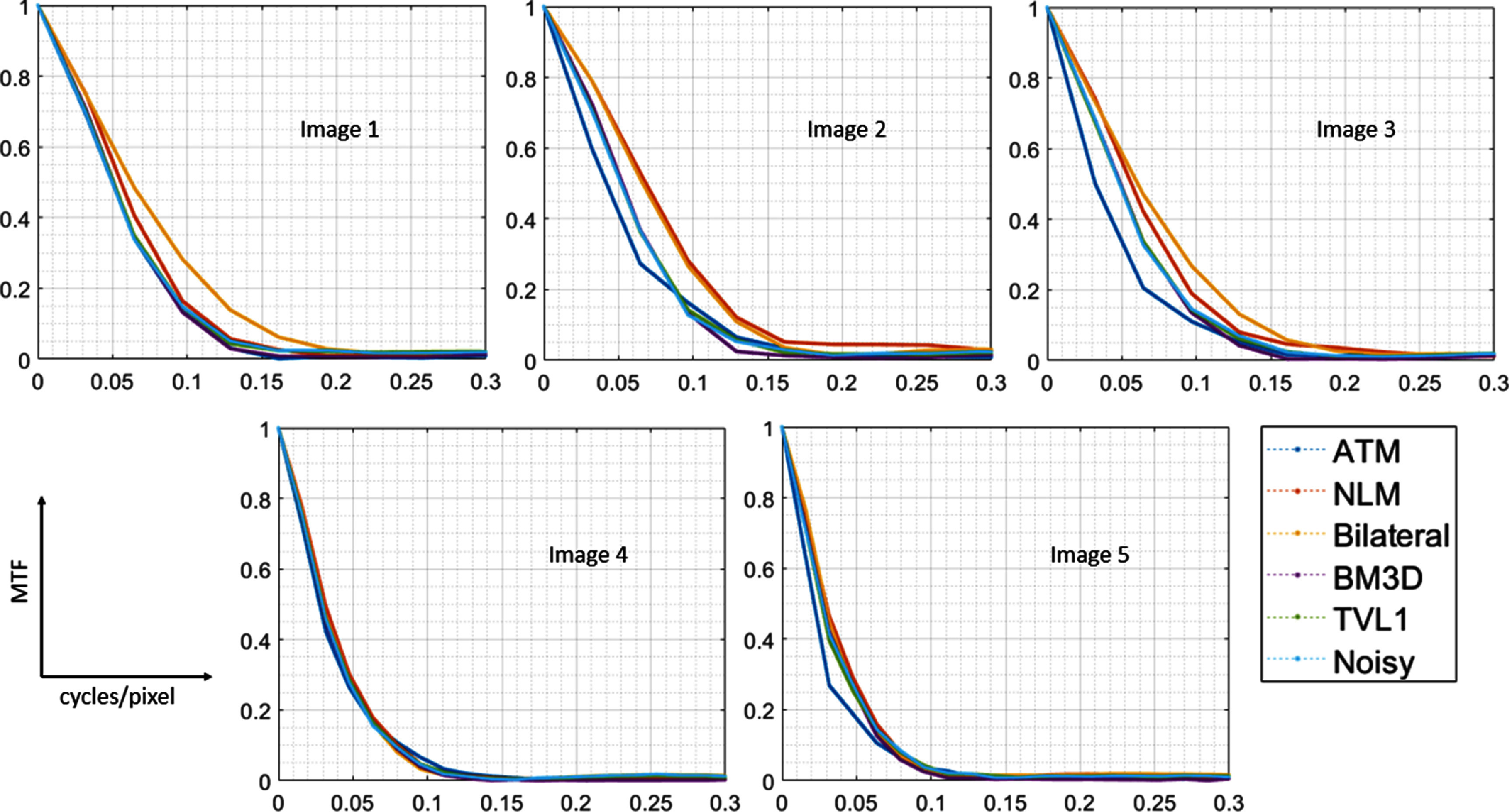
MTF plots for all images with the filters applied.

**Figure 10.**
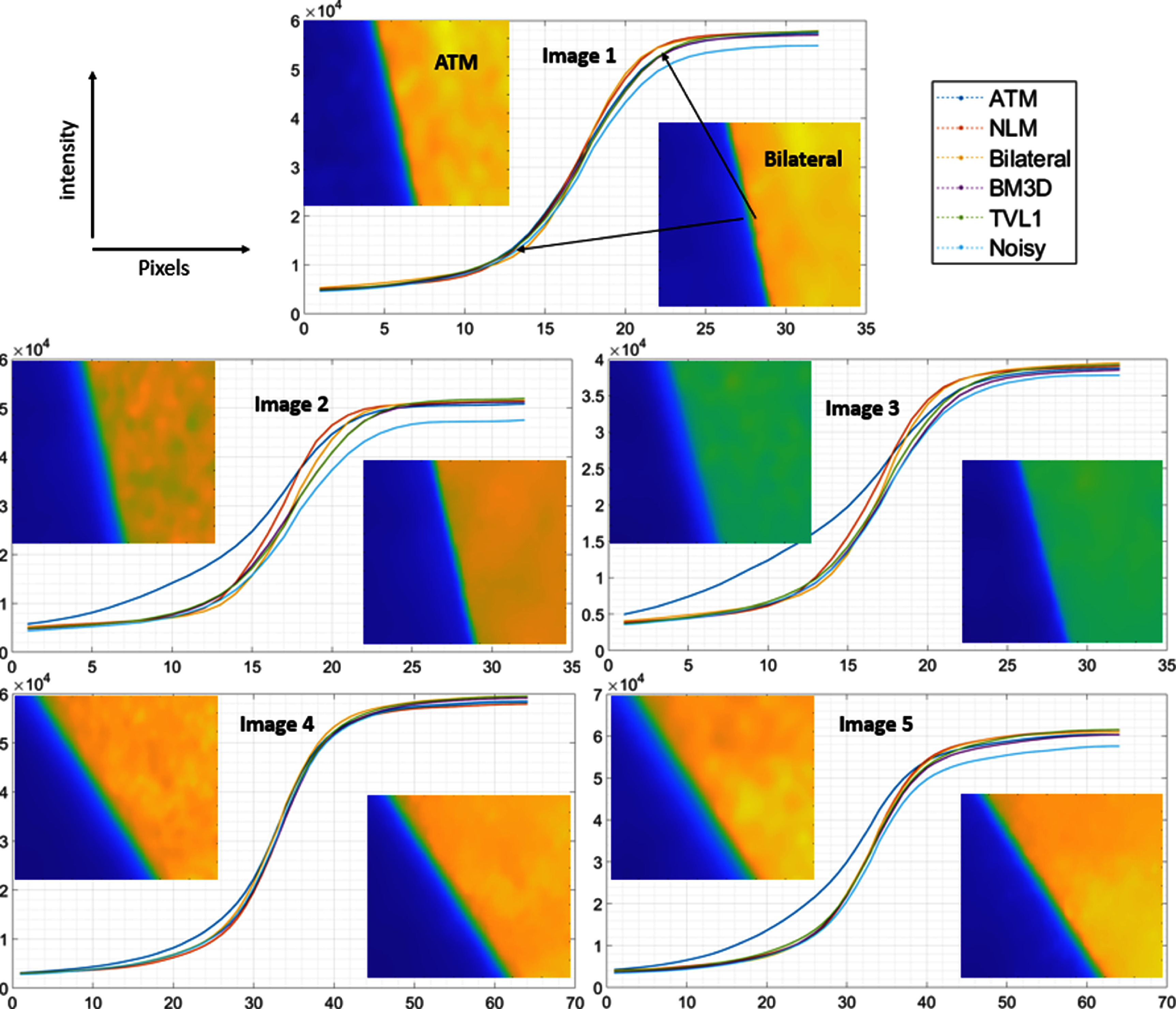
ESF plots for the noisy and filtered images. Zoomed images for the edges for the most blurred (ATM) and sharpest (bilateral) edge are included on the plot for visualization.

Both the rise distance and the MTF were estimated for the noisy and filtered images as described in the methods. The MTF plots are depicted in figure 9 for each image while the ESF is shown in figure 10.

**Figure 11.**
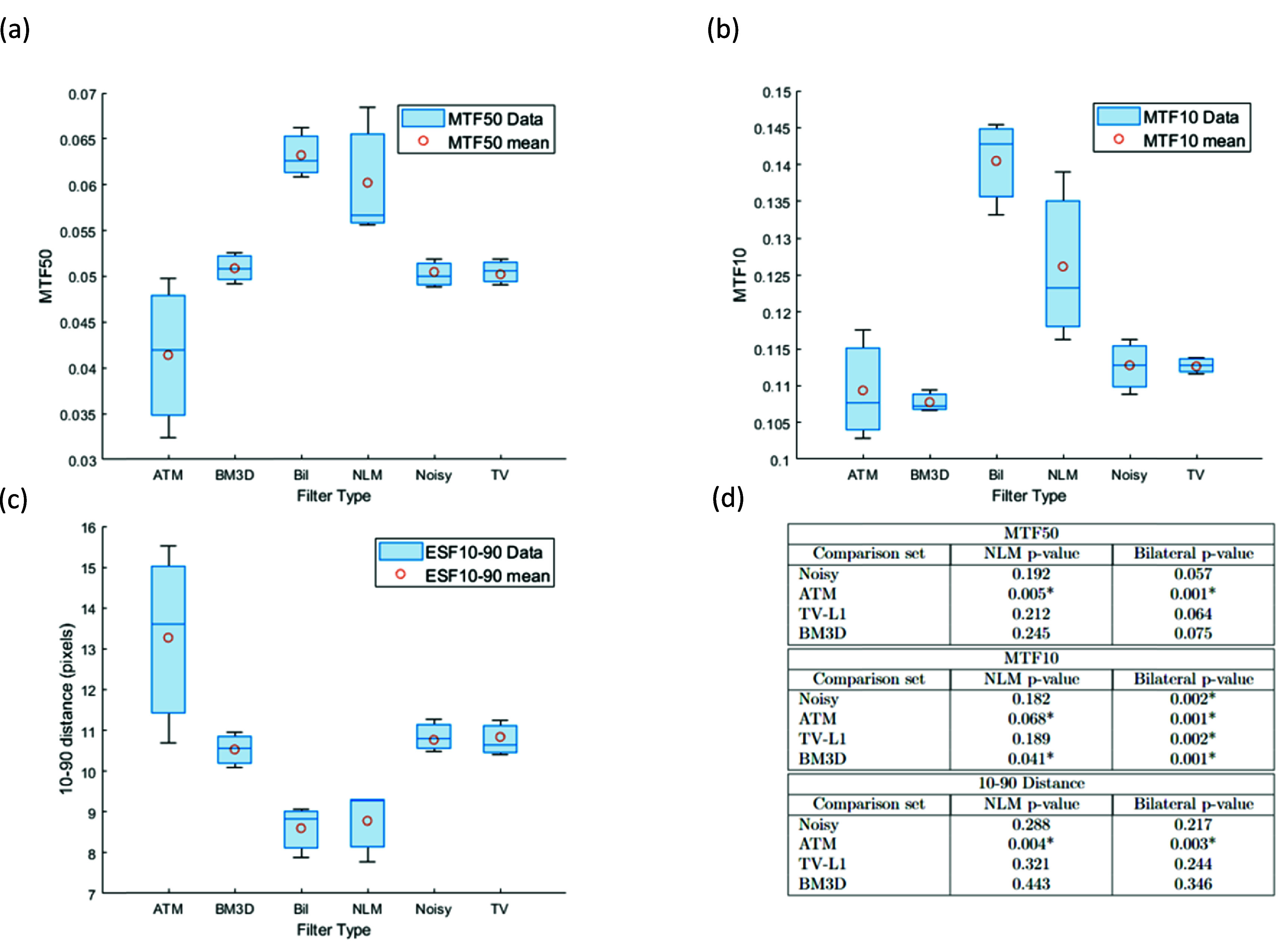
Edge sharpness metrics for each filtered image. (a) and (b) Shows larger MTF values for the bilateral and NLM filters over the noisy image implying that they enhance the edge sharpness. BM3D and TV show minimal edge blurring while the ATM filter produces blurrier edges. These results are corroborated by (c) where the rise distance decreases for the bilateral and NLM filter, remains roughly constant for the BM3D and TV filters, and increases for the ATM filter. (d) Shows the result of a multiple comparison test using the NLM and bilateral filters as the control groups. The star (*) highlights differences that are statistically significant according to a 95% confidence criteria.

From these plots, the MTF50, MTF10, and 10-90 rise distance were calculated. According to these metrics, the sharpness increased for both the NLM and bilateral images because the large noise levels in the original image contribute to blurring of the edges. Although the denoising filters are expected to reduce the inherent sharpness of the image, the noise reduction is sufficient to improve the edge sharpness overall. Conversely, the ATM algorithm produced an increase in edge blurring which can be observed by the increased 10-90 rise distance and decreased MTF50 and MTF10 in figure 11. Tables 1 and 2 list the values calculated for each metric. For the plot depicted in figure 11, only images 1-3 were analyzed as images 4 and 5 exhibited a slower edge transition due to how the image was setup with the phantom. Although not depicted, these images showed a similar trend as can be deduced from tables 1 and 2.

**Table 1.** MTF50 and MTF10 data for the 25MU images. These values mark the frequency in cycles per pixel where the MTF plot crosses the 50% and 10% points. Due to the difference in edge sharpness caused by the image setup, only the first three data points are used to form the plot in figure 11.

MTF50 (cycles/pixel)					
Filter	Image 1	Image 2	Image 3	Image 4	Image 5

Noisy	0.050	0.052	0.049	0.030	0.027
TVL1	0.051	0.052	0.049	0.029	0.026
NLM	0.056	0.068	0.057	0.031	0.030
ATM	0.050	0.042	0.032	0.028	0.022
Bilateral	0.063	0.066	0.061	0.030	0.029
BM3D	0.051	0.053	0.049	0.029	0.028

MTF10 (cycles/pixel)					

Filter	Image 1	Image 2	Image 3	Image 4	Image 5

Noisy	0.113	0.109	0.116	0.078	0.075
TVL1	0.112	0.113	0.114	0.078	0.075
NLM	0.116	0.139	0.123	0.081	0.075
ATM	0.108	0.118	0.103	0.083	0.066
Bilateral	0.145	0.113	0.143	0.076	0.072
BM3D	0.107	0.107	0.109	0.078	0.070

**Table 2.** The 10%–90% distance is larger for images with worse resolution. As stated for table 1, only the first three data points are used to form the plot in figure 11.

10-90 distance (pixels)					
Filter	Image 1	Image 2	Image 3	Image 4	Image 5

Noisy	10.78	10.47	11.25	18.29	20.39
TVL1	10.65	10.39	11.25	18.45	20.74
NLM	9.29	7.76	9.28	16.96	18.10
ATM	10.70	13.60	15.50	20.02	24.89
Bilateral	8.83	7.89	9.07	17.51	17.64
BM3D	10.56	10.08	10.94	18.69	19.70

A comparison between the four algorithms favors the NLM and bilateral filters as they exhibit the shortest rise distance as well as the largest MTF50 and MTF10 frequencies Larger MTF50 and MF10 values correlate to the presence of higher frequencies in the image which help form sharper edges. Multiple comparison analysis was conducted using the Tukey honestly significant difference procedure. The image sharpness metrics for both the NLM and bilateral filters showed statistically significant differences from the values of several of the other filters as shown in figure 11(d).

### 3.3. Radial frequency

**Figure 12.**
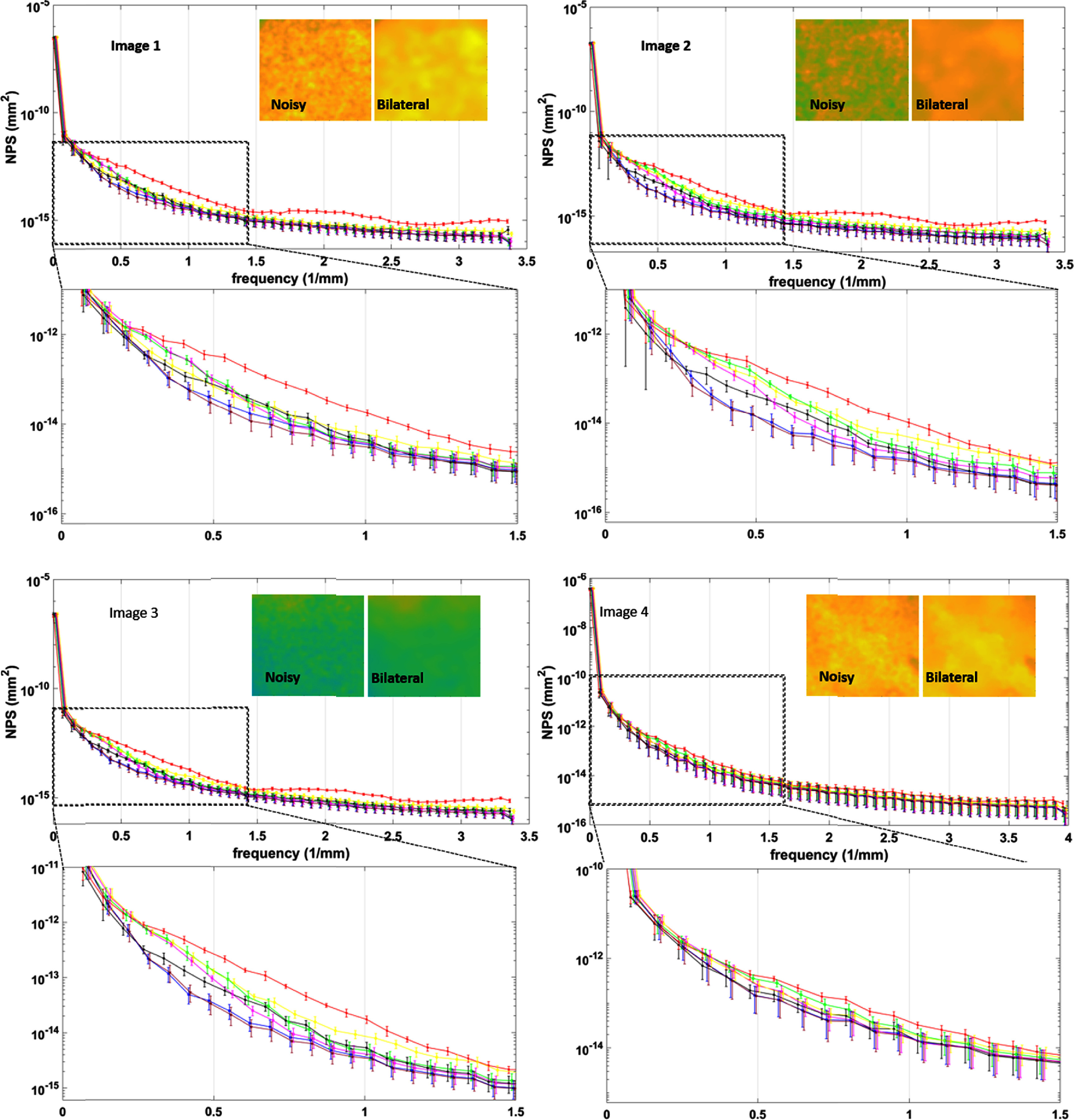
NPS plots of the unfiltered, TV-L1, NLM, ATM, bilateral, BM3D and ground truth images. Error is calculated as the standard deviation of the NPS data points from the set of ROIs. The NLM and bilateral curves show a greater reduction in noise intensity in the low frequency range on average. Zoomed visualizations of the noisy and bilateral images are included to show the improvement in image homogeneity. Note that the data points of different filters are offset to allow for easier visualization.

The radially averaged NPS was obtained for each filtered image along with the noisy image as described in section 2.7.3. This analysis is displayed in figure 12. Figure 12(a) displays the NPS for the noisy data along with the data produced with the five denoisers. Error bars were calculated for the NPS curves by computing the standard deviation of the ROI at each frequency point. The NLM and bilateral filters produced the lowest NPS values on average for 0–1.5 mm^−1^ frequencies. It should be noted that higher frequencies are less perceptible to the eye which is why figure 12 focuses on the lower frequency regions of the plots.

This reduction of the NPS values for the NLM and bilateral filters is below the NPS calculated for the ground truth image, suggesting that these filters over smooth the image. This suggests that these algorithms lead to a loss of image detail. This should be considered when applying these filters.

## 4. Discussion

Cherenkov imaging has high potential for dosimetry and radiation incident detection, but the inhomogeneity of light detection due to the high levels of noise and resulting mottle can make proper skin dose determinations difficult. This study sought to use static image denoising algorithms to improve the accuracy of cumulative Cherenkov images. This type of post-processing could be included in the Cherenkov imaging software to provide clinics with cleaner cumulative images of surface Cherenkov emission. Although not explored in this analysis, other denoising algorithms exist which may also be worth including, such as wavelet-based denoising or total variation with an L2 norm.

Compared to the unfiltered Cherenkov data, the NLM, bilateral, and TV-L1 denoising approaches provided an 19.1%, 19.0%, and 17.4% increase in PSNR on average across the analyzed 25MU images. The ATM and BM3D filters, on the other hand, produced only a 10.9% and 12.13% increase, respectively, on average. The blurring imposed on the image by the NLM and bilateral algorithms was sufficiently countered by the denoising improvement as the MTF10 and MTF50 increased by 24.6% and 25.8%, respectively, for the bilateral images and by 11.8% and 19.9%, respectively, for the NLM images.

It should be recognized that there are some limitations to this study. As stated in the description of the BM3D filter, the PSD was assumed to be Gaussian which may not perfectly represent the noise present in Cherenkov images. Investigation into the application of blind-noise BM3D methods for Cherenkov could prove beneficial (Jia *et al* 2022). Additionally, while the NLM approach showed great denoising results, advancements to this algorithms have recently been published that incorporate gradient structure similarity when determining weights (Kong *et al* 2024). Employing this algorithm could further enhance denoising performance for future work. Another limitation concerning the NLM and ATM filters relates to the need to select default values for the various parameters. Values were selected based on simple algorithm testing, however, multidimensional optimization could have provided better results, although it is likely that only minimal improvements would be achieved while being computationally expensive. The TV-L1 algorithm, although able to provide excellent smoothing and edge preservation, is limited by non-linearity artifacts and outputs that appear artificial, which result in PSNR values falling below both the NLM and bilateral approaches.

There are also limits concerning the variability in Cherenkov images as only two phantoms were available. While these phantoms are good approximations of human tissue, they are not perfect replicas. Additionally, the preservation of blood vessels in the image is not assessed as these phantoms do not contain such structures. Despite these limits, the dominant sources of noise, such as read noise, photon shot noise, and stray x-ray photons, are present regardless of the subject being imaged. Therefore, we believe this analysis to be largely applicable to actual patient data.

It can be difficult to filter the single frame data due to the lack of signal, but a few strategies might prove beneficial in future work. By lowering the frame rate to accept a larger number of photons per frame, the NLM or bilateral algorithm could be applied to denoise a lower framerate video sequence, ideally in real time. A moving temporal window approach could also be applied to maintain a higher frame rate. In future work, these algorithms could be applied to dynamic-cumulative Cherenkov images of non-static beam therapies at each control point of the treatment, which will be particularly valuable for any treatment plans with beams that move over the course of the treatment such as intensity modulated radiation therapy and volumetric modulated arc therapy (VMAT). Due to the nature of VMAT procedures where the gantry is constantly rotating and changing the shape of the multileaf collimators, there is often less dose delivered at each entry point of the patient as there would be in static beam therapy. Therefore, fewer Cherenkov photons reach the sensor for each body region enhancing the noise levels detected. These denoising approaches could be beneficial for improving this data.

Additionally, Cherenkov images generated from light exiting the skin of patients with a dark skin tone could see improvement. The higher a patient’s melanin levels, the more their skin attenuates the Cherenkov light produced below the skin surface. This results in images exhibiting fewer statistics and larger noise levels compared to images generated off patients with lighter skin. The NLM or bilateral algorithms could be applied along with a series of processing steps to help reduce problems like this where something about the tissue limits the detected light signal. While the algorithm may diminish noise, it remains to be seen how it will affect the detection of small details such as blood vessels typically visible in external beam Cherenkov imaging.

Incorporation of any of these filters into the clinical workflow will be challenging due to the computationally intensive processing necessary to filter each image in addition to any parameter optimization as was used for this study. Filtering on chip may not be feasible and the denoised image may not be available in real time for the physician to observe. To cut back on computation time, ideal parameters would need to be pre-selected for a given room lighting condition as well as patient skin tone and treatment site. Filtering would take place after the treatment is delivered. Another solution could be to train a machine learning model using these filters as reference which could potentially be implemented in the clinical system.

Most modern denoising algorithms employ convolution neural networks which often rely on ground truth images to guide the model. Thakur *et al* (2023) highlights several models employed in medical imaging. Specifically, CNN-DMRI (CNN for denoising magnetic resonance images) and DnCNN (denoising CNN) are used for MR denoising and TS-RCNN (two stage residual CNN) and CVMIDNet (complex valued medical image denoiser network) are often used to denoise CT images (Thakur *et al* 2023). Additionally, Tian *et al* (2020) demonstrated CNN denoising with batch renormalization (Tian *et al* 2020a) and Choi *et al* (2019) demonstrated low dose CT denoising using a generative adversarial network (Choi *et al* 2019). An extensive list of other machine learning denoising methods can be found in the deep learning overview by Tian *et al* (2020) (Tian *et al* 2020b).

These models could be modified to denoise individual Cherenkov frames but will require a large database of phantom and patient images with ground truth data of sufficient quality. While such high-quality images can be obtained for phantom irradiations by performing long linac exposures, ground truth of the same quality cannot be obtained for patient images due to the increased radiation exposure they would require. However, as highlighted in this work, noisy cumulative images can be filtered to increase the PSNR. These filtered images can act as the ground truth in a machine learning denoising workflow and will be the focus of future Cherenkov denoising improvements. A similar approach has been used in PET imaging using an imaged optimally denoised with a bilateral filter as the ground truth for training a CNN (Maus *et al* 2024). Additionally, transfer learning techniques could be applied to further improve denoising results. Models trained on large data sets that contain similar noise profiles to Cherenkov data (such as simulated images or other low light CMOS images) could be fine-tuned with Cherenkov phantom images in a method similar to the process described in the 2020 study by Hegazy *et al* on dental CT denoising (Hegazy *et al* 2020). With these techniques, high quality clinical Cherenkov data may not be needed to effectively train a denoising model.

## 5. Conclusion

This study has shown that image denoising algorithms can be included as another step in the network of Cherenkov post-processing. The NLM and bilateral filters provided the greatest increase in PSNR while also maintaining sharpness of the edge while the TV-L1 filter also provides comparable results. Both the superior PSNR and edge sharpness suggest that the NLM and bilateral filters are the best for the use of Cherenkov post-processing. Future analysis should be directed toward applying this knowledge to denoise cumulative images to act as ground truth for machine learning denoising of individual frames as discussed above.

## Data Availability

• The archived version of the alpha (adaptive) trimmed mean (ATM) code described in the manuscript can be freely accessed through Code Ocean https://codeocean.com/capsule/2512793/tree. • The above link also contains the MATLAB code of the algorithm implementation and analysis along with the code used to produce the plots. • The MTF ImageJ plugin can be found using the following link https://imagej.net/ij/plugins/se-mtf/index.html. • The archived version of the total variation code (TV-L1) described in the manuscript can be freely accessed through the MATLAB Central File Exchange www.mathworks.com/matlabcentral/fileexchange/57604-tv-l1-image-denoising-algorithm. • The archived version of the non-local means (NLM) code described in the manuscript can be freely accessed through the MATLAB Central File Exchange www.mathworks.com/matlabcentral/fileexchange/38200-fast-non-local-mean-image-denoising-implementation. • The archived version of the block matching 3D (BM3D) code described in the manuscript can be freely accessed through the Tampere University of Technology Department of Signal Processing https://webpages.tuni.fi/foi/GCF-BM3D/index.html. • Data will be shared by the author upon request to jehallett@wisc.edu The data cannot be made publicly available upon publication because they are not available in a format that is sufficiently accessible or reusable by other researchers. The data that support the findings of this study are available upon reasonable request from the authors.
